# Subversion of RAB5-regulated autophagy by the intracellular pathogen *Ehrlichia chaffeensis*

**DOI:** 10.1080/21541248.2017.1332506

**Published:** 2017-07-05

**Authors:** Yasuko Rikihisa

**Affiliations:** Department of Veterinary Biosciences, The Ohio State University, Columbus, OH, USA

**Keywords:** autophagy, BECN1, class III PtdIns3K, *Ehrlichia chaffeensis*, endosome, Etf-1, infection, obligatory intracellular, RAB5, type IV secretion

## Abstract

Intracellular pathogens often exploit RAB functions to establish a safe haven in which to survive and proliferate. *Ehrlichia chaffeensis*, an obligatory intracellular bacterium, resides in specialized membrane-bound inclusions that have early endosome–like characteristics, e.g., resident RAB5 GTPase and RAB5 effectors, including VPS34 (the catalytic subunit of class III phosphatidylinositol 3-kinase), but the inclusions lack late endosomal or lysosomal markers. Within inclusions, *Ehrlichia* obtains host-derived nutrients by inducing RAB5-regulated autophagy using *Ehrlichia* translocated factor-1 deployed by its type IV secretion system. This manipulation of RAB5 by a bacterial molecule offers a simple strategy for *Ehrlichia* to avoid destruction in lysosomes and obtain nutrients, membrane components, and a homeostatic intra-host-cell environment in which to grow.

## Introduction

RAB GTPases localize to distinct cellular membrane compartments and regulate multiple steps in eukaryotic vesicle trafficking including vesicle budding and invagination, vesicle tethering, and membrane fusion.^1,2^ RAB5 is found in nascent phagosomes and early endosomes that lack the microbicidal capacity required to kill invading pathogens; this requires subsequent maturation to late endosomes and subsequent fusion with lysosomes. Consequently, several intracellular pathogens subvert RAB5 functions. For example, *Mycobacterium tuberculosis* localizes to the RAB5-positive endocytic compartment by reducing the amount of phosphatidylinositol 3-phosphate (PtdIns3P) in the phagosomal membrane, thereby interfering with phagosome maturation.^3,4^ *Listeria monocytogenes* GAPDH binds and ADP-ribosylates RAB5A and impairs GDP/GTP exchange on this GTPase, thereby blocking phagosome maturation.^5^ The *Salmonella* type III secretion system effector SopE (a GDP/GTP exchange factor mimic) activates RAB5 to trigger entry into host cells and promote fusion with early endosomes,^6^ whereas SopB (a phosphoinositide phosphatase) recruits RAB5 and its effector VPS34 to promote maturation of *Salmonella*-containing vacuoles.^7^

*Ehrlichia chaffeensis* is a tick-borne obligatory intracellular bacterium that causes human monocytic ehrlichiosis, an emerging life-threatening infectious disease in the US and other parts of the world.^8^ Upon entry into host cells via receptor-mediated endocytosis,^9^ *Ehrlichia* resides in specialized membrane-bound inclusions that provide sanctuary against host innate-immune microbicidal mechanisms and a means to acquire host cell–derived nutrients. These intracellular inclusions have early endosome–like characteristics, including transferrin receptor, transferrin, vacuolar-type H^+^-ATPase, and the small GTPase RAB5 and its effectors EEA1 (early endosome antigen 1), VPS34, and Rabankyrin-5, but they lack late endosomal or lysosomal markers or NADPH oxidase components.^8^^,^^10^^,^^11^ *E. chaffeensis* infection is dependent on RAB5 and RAB5-regulated trafficking to establish *E. chaffeensis* inclusions, indicating that the recruitment and retention of RAB5-containing endosomes are necessary for subsequent expansion of the inclusions and pathogen replication.^11^ RABs cycle between GTP-bound “active” and GDP-bound “inactive” conformational states. The exchange of GDP for GTP in RABs is catalyzed by a guanine nucleotide exchange factor (GEF), whereas GTP hydrolysis is facilitated by a GTPase-activating protein (GAP).^12^ Overexpression of RAB5A^S34N^ (dominant-negative RAB5A/RAB5A-DN, a GDP-bound form of RAB5 that sequesters GEF for RAB5 and thus prevents RAB5 activation) or the RAB5-specific GAP prevents *E. chaffeensis* infection,^11^ indicating that GTP-bound RAB5 is required for *E. chaffeensis* infection.^11^

One fundamental aspect of microbial pathogen virulence is “nutritional virulence,"^13^ i.e., the ability to acquire nutrients for pathogen proliferation in competition with host cells and possibly other microbes. Because *E. chaffeensis* has a small genome (1.176 Mb) and relatively few protein-coding genes that are needed for biosynthesis of amino acids and intermediary metabolites,^8^ the pathogen relies heavily on host-derived nutrients. Although the host-cell cytoplasm is rich in these nutrients, the ehrlichial inclusion membrane is essentially impermeable to nutrient flow, considering the fact that the inclusion maintains a weakly acidic intraluminal pH.^10^ It is also unlikely that varieties of active transporters are synthesized and assembled on the inclusion membrane to import host nutrients during ehrlichial replication. Because *Ehrlichia* cannot replicate or even survive outside of a eukaryotic cell, this mechanism must ensure host-cell survival until *Ehrlichia* have undergone sufficient replication. Similar limitation is also found in a tick-borne obligatory intracellular bacterium, *Anaplasma phagocytophilum*, in the family Anaplasmataceae to which *E. chaffeensis* belong. *A. phagocytophilum* replicates in neutrophils in MAP1LC3/LC3 (microtubule associated protein 1 light chain 3; a mammalian ortholog family of yeast Atg8)-decorated early autophagosomes that are segregated from the endosomal and lysosomal pathway, thereby gaining access to host cytosolic nutrients while escaping autolysosomal degradation.^14-16^ Because *E. chaffeensis* resides in the intracellular compartment distinct from *A. phagocytophilum*,^8^^,^^10^^,^^11^ and the compartment lacks LC3, out hypothesis was that *E. chaffeensis* species have evolved a unique molecular mechanism to acquire host-cell nutrients.

### Autophagy is required for Ehrlichia proliferation

Autophagy is an essential and highly regulated eukaryotic cellular homeostatic process that sequesters and digests/recycles intracellular components,^17,18^ and autophagy is an important innate immune response to kill intracellular bacteria such as *Salmonella, Shigella, Listeria*, and *Mycobacterium*^19-23^ Induction of autophagy by rapamycin promotes intracellular killing of *Salmonella* and *Mycobacterium*,^23,24^ and inhibition of autophagy by the PtdIns3K inhibitor 3-methyladenine promotes the survival of intracellular mycobacteria.^23^ In contrast to these bacteria, the intracellular replication of *E. chaffeensis* is enhanced by rapamycin and strongly inhibited by 3-methyladenine.^11^ BECN1 (beclin 1; mammalian ortholog of yeast Vps30/Atg6) is an essential component of the class III PtdIns3K complexes that activate canonical autophagy.^25^ A study using the cell-permeable and potent inhibitor of autophagy, spautin-1, BECN1 siRNA, or mouse bone marrow–derived macrophages from *atg5^flox/flox^-Lyz2-Cre* mice (in which *Lyz2* promoter-driven *Cre* is used for myeloid cell–specific *Atg5* knockout) point to autophagy as not only enhancing ehrlichial infection but also being required for replication.^11^ Thus, *E. chaffeensis* does not simply escape innate immune clearance via cellular autophagy but rather takes advantage of autophagy for its proliferation.

### Autophagy is induced by bacterial type IV secretion effectors

The best-known mechanisms of autophagy induction are those induced by amino-acid starvation initiated by the activation of ULK, which is mainly regulated by the MTOR kinase complex or by PRKA/AMPK (protein kinase, AMP-activated) independently of MTOR activity.^26,27^ However, *E. chaffeensis* induces autophagy independently of MTOR, ULK1, and PRKA/AMPK.^11^ Upon their ubiquitination, intracellular aggregated proteins, overabundant proteins, and certain microbial proteins are selectively targeted to autophagosomes for degradation through the ubiquitin- and LC3-binding protein SQSTM1/p62 (sequestosome 1).^28,29^ Certain proteins of intracellular vacuoles that contain *Salmonella, Streptococcus*, or *Legionella* are ubiquitinated, and subsequent binding of p62 delivers the ubiquitin-marked vacuoles to autolysosomes for degradation.^30-32^ However, proteins of inclusions containing *E. chaffeensis* are not ubiquitinated as monoclonal antibody FK2 that recognizes both mono- and poly-ubiquitinated proteins did not label the inclusions, and the extent of overall (cellular/bacterial) protein ubiquitination is not increased in infected cells by western blot analysis using FK2 and monoclonal antibody FK1 that recognizes poly-ubiquitinated proteins.^11^ How, then, does *Ehrlichia* induce autophagy?

*E. chaffeensis* has a functional type IV secretion system^33^ that mediates the transport of bacterial molecules, referred to as effectors/substrates, across the bacterial and host-cell membranes into the cytoplasm of host cells using bacterial-derived energy.^34^ One of the type IV secretion system effectors, Etf-1 (*Ehrlichia* translocated factor-1), interacts with BECN1, VPS34, and RAB5^11^ and activates class III PtdIns3K, which induces autophagy independently of amino-acid or ATP depletion.^11^

### RAB5-regulated autophagy

RAB5 regulates early endosome maturation to late endosomes, thereby coordinating degradation of extracellular components. RAB5 also regulates degradation of intracellular components by coordinating the fusion of LC3-decorated autophagosomes with late endosomes to form intermediary compartments before fusion with lysosomes.^35^ Furthermore, in an increasing number of cellular processes, RAB5 has been found to regulate autophagy upstream of LC3 conjugation,^36-38^ which is called as “RAB5-regulated autophagy.”^38^ The exact factors that induce this type of autophagy vary widely depending on the cellular circumstances, but they are conceptually similar: constitutively active RAB5 induces autophagy by binding to VPS34, which binds to BECN1, an essential component and master regulator of class III PtdIns3K complex. On the other hand RAB5-DN cannot bind VPS34, thus it not only cannot induce autophagy, but also inhibits autophagy.

Etf-1 induces RAB5-regulated autophagy. VPS34 and BECN1 interact with Etf-1 only in the presence of GTP-bound (active) RAB5, but not GDP-bound RAB5.^11^ Ectopic expression of Etf-1 in mammalian cells activates class III PtdIns3K and induces the biogenesis of numerous ATG5-containing structures (autophagosome precursors) and LC3-containing autophagosomes.^11^ Deconvolution fluorescence microscopy has revealed that Etf-1–containing puncta line the inner side of the enlarged GFP-tagged constitutively active RAB5 endosomes when these 2 molcules are co-expressed in mammalian cells.^11^

The details of RAB5-regulated autophagy have not been fully elucidated because this type of autophagy is not easily followed in the well-studied mammalian-cell systems in which autophagy is induced by amino-acid or ATP starvation. However, RAB5-regulated autophagy has been reported in several systems in which autophagy is induced independently of the activation of MTOR signaling systems, noted as follows.^11^^,^^36^^,^^38^ First, ectopic expression of RAB5A-DN or treatment of cultured cells with 3-methyladenine blocks the progression of early ATG5-positive phagophores to form LC3-positive autophagosomes.^38^ Second, upon growth-factor restriction, the class 1A PtdIns3K catalytic subunit β (PIK3CB/p110β) dissociates from growth factor receptor and binds RAB5.^36^ This interaction blocks the binding of the RAB5 GAP, PIK3R1/p85α (class 1A PtdIns3K regulatory subunit 1) to RAB5, which consequently increases the amount of GTP-bound RAB5 and enhances the RAB5-VPS34 interaction to promote autophagy.^36^ Third, the hepatitis C virus protein NS4B forms a complex with RAB5 and VPS34 and promotes RAB5-GTP–induced autophagy, which is required for virus replication.^37^ Finally, eukaryotic proteasomes can only very poorly cleave (if at all) polyglutamine sequences such as polyglutamine tracts in huntingtin protein.^39^ The expansion of polyglutamine tracts in huntingtin causes aggregation of polyglutamine-containing peptides, leading to the neurodegenerative genetic disorder Huntington's disease.^40^ The protein huntingtin binds RAB5 via the RAB5 effector F8A1/HAP40 (coagulation factor VIII–associated 1) and is degraded during autophagy.^41^ Interestingly, Etf-1–induced autophagy also clears an aggregation-prone mutant huntingtin protein in a class III PtdIns3K–dependent manner,^11^ although whether the HAP40 adaptor is involved in this process is unknown.

In addition to these examples, the endoplasmic reticulum–localized transmembrane protein EMC6 (endoplasmic reticulum membrane protein complex subunit 6) interacts with both RAB5A and BECN1 to induce autophagosomes;^42^ notably, EMC6 colocalizes with DFCP1, a marker of the omegasome (amino-acid starvation–induced precursor of isolation membrane). Ypt53, an isoform of RAB5 in yeast, is upregulated significantly under nutrient stress to maintain both Golgi–vacuole trafficking and vacuolar hydrolase activity.^43^ Overall, published data suggest a broad involvement of RAB5 in autophagy, including amino-acid starvation–induced autophagy. In addition to RAB5, other RABs may be involved in autophagy regulation; several other RABs as well as a group of TBC (tre2-bub2-cdc16) domain–containing RAB GAPs and non-TBC RAB GAPs have been shown to regulate autophagosome formation (for review see refs. 44, 45).

### RAB5-regulated autophagy as a bacterial nutrient acquisition mechanism

**Figure 1.**
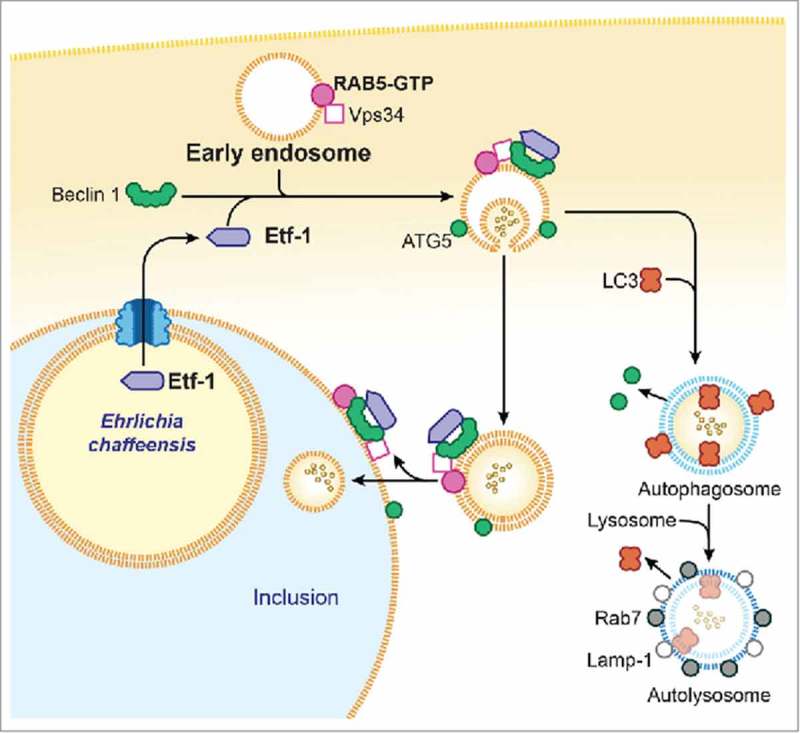
Proposed model for Etf-1-mediated autophagy fueling *E. chaffeensis* growth. Secreted Etf-1 interacts with RAB5, VPS34, and Beclin 1 to induce complex formation and localize to the ATG5-positive “precursor of preautophagosomes.” If not fused with *E. chaffeensis* inclusions, Etf-1 autophagosomes mature to autolysosomes to generate cytosolic nutrients (∘∘) (right side). When nascent Etf-1 preautophagosomes fuse with *E. chaffeensis* inclusions, they deliver captured cytosolic nutrients to the inclusions where lysosomal fusion is blocked (left side).

Etf-1–induced autophagy releases host-cell small-molecule catabolites into the host cytoplasm without actually starving host cells. An additional advantage of bacterial effector–induced autophagy is that it creates more host cytoplasmic space for bacterial growth without initially harming host cells. During the exponential growth stage of *E. chaffeensis*, the concentrations of free/cytoplasmic l-glutamine and l-glutamate in infected human monocytes are notably increased, making them available for ehrlichial growth.^11^ Indeed, host cell–preincorporated radioactive amino acids can be readily taken up by *E. chaffeensis* in an autophagy-dependent manner, and the cytoplasmic autophagy cargo protein human GAPDH^46^ can be delivered to *E. chaffeensis* inclusions.^11^ However, how do inclusion-confined *E. chaffeensis* acquire host cytoplasmic molecules? Although the classical view has been that the autophagic and endocytic pathways converge at the phagolysosomal and lysosomal levels, autophagosomes have also been found to undergo fusion with components of the early endocytic pathway.^35^^,^^47^^,^^48^ RAB5, VPS34, and Etf-1–containing vesicles are targeted to already-established *E. chaffeensis* inclusions, suggesting the recruitment of RAB5 endosomes during *E. chaffeensis* replication and expansion of the inclusion. Thus, *E. chaffeensis* inclusions can be considered as large amphisomes formed by fusion of early endosomes and early autophagosomes because several early-endosome markers, Etf-1, and the early autophagosome marker ATG5 (but not LC3) are present on the membrane of *E. chaffeensis* inclusions.^11^ Because Etf-1–induced autophagosomes can capture host-cell cytoplasmic nutrients, if newly nucleated autophagosomes fuse with ehrlichial inclusions before their maturation into LC3-positive autophagosomes or autolysosomes, these autophagosomes can deliver the nutrients to the inclusions. Another possibility is that Etf-1–induced autophagosomes (vesicles) in infected cells are already amphisomes formed by fusion of early endosomes because these vesicles that are docked to inclusions also contain RAB5, VPS34, ATG5, and Etf-1 but not LC3, as does the inclusion membrane itself. Once these small amphisomes fuse with ehrlichial inclusions, they deliver host cytoplasmic nutrients into the inclusion lumen. Endosomes, themselves may also facilitate cytosolic nutrient delivery to ehrlichial inclusions. Multivesicular bodies (MVBs), which are morphologically distinct endosomes internally accumulate small membrane vesicles (60 to 80  nm) that contain cytoplasmic cargo molecules.^35,49^ Morphological evidence suggests that MVBs are the main endocytic fusion partner of autophagosomes, forming the amphisome.^35,50^ class III PtdIns3K and PtdIns3P are essential for the generation of the internal vesicles of MVBs, and RAB5 promotes the biosynthesis of PtdIns3P on endosomes^51^ and phagosomes.^52^ PtdIns3P accumulates within MVBs.^53^ Thus, most labeling of 2 × FYVE-GFP that binds to PtdIns3P is associated with the internal membranes of MVBs, which contain only small amounts of the markers for late endosomes and lysosomes.^53^ Results with 2 × FYVE-GFP–transfected cells has revealed large amounts of intraluminal PtdIns3P in large vesicles docked to *E. chaffeensis* inclusions, suggesting that these vesicles are MVBs.^11^ If MVBs fuse with ehrlichial inclusions, then host cytoplasmic nutrients can be effectively delivered to inclusions. Fig. 1 presents our proposed model of RAB5-regulated autophagosome nucleation and subversion by Etf-1 in *E. chaffeensis*–infected cells.

## Conclusions

Intracellular pathogens such as bacteria and virus can utilize a recently defined pathway, namely RAB5-regulated autophagy, to their advantage. The possibility exists that RAB5-regulated autophagy may be induced by other unidentified eukaryotic, bacterial, and viral proteins that can interact with RAB5 or RAB5 regulatory proteins. Perhaps the most salient feature of RAB5-regulated autophagy in ehrlichial infection is that preexisting host-cell components and signaling pathways are repurposed in a way that is independent of other signaling pathways by using a bacterial molecule. The bacterial molecule can be used to predictably induce autophagy-related signaling to clear the abnormal intracellular accumulation of toxic molecules, damaged organelles, and pathogenic agents. Currently the only therapy for human monocytic ehrlichiosis is the broad-spectrum antibiotic doxycycline, which is effective only if initiated early, and no vaccine is available. Mechanistic understanding of nutritional virulence of *E. chaffeensis* will provide important scientific basis for novel anti-*E. chaffeensis* therapy and preventive measures.
